# Impact of density of schistosomal antigen expression in urinary bladder tissue on the stratification, cell type, and staging, and prognosis of carcinoma of the bladder in Egyptian patients

**DOI:** 10.1186/1750-9378-9-21

**Published:** 2014-06-26

**Authors:** Mohamed Wishahi, Ahmed Zakarya, Olfat Hamamm, Mohamed Abdel-Rasol, Hisham Badawy, Hossam Elganzoury, Mohamed Ismail, Amr Elkhouly, Ahmed Meheina

**Affiliations:** 1 Department of Urology, Theodor Bilharz Research Institute, Kornish El Nil, 12343, Embaba/Giza, P.O.B. 30, Cairo, Egypt; 2 Department of Pathology, Theodor Bilharz Research Institute, Cairo, Egypt; 3 Department of Urology, Faculty of Medicine, Cairo University, Cairo, Egypt

**Keywords:** Schistosomiasis, Infection, Urinary bladder, Urothelial carcinoma, Squamous cell carcinoma, Cancer prognosis, Endemic diseases, Neglected diseases, Bilharziasis, Schistosomal antigen, Monoclonal antibodies, Tumour marker

## Abstract

**Background:**

Infection with urinary schistosomiasis and its severity are oncogenic factors for developing carcinoma of the bladder, whether it is urothelial carcinoma (UC) of a transitional cell type (TCC) or non-urothelial of squamous cell carcinoma (SCC). In UC it is not defined whether it is schistosomal or not. This led to controversial results in expression of tumour markers, tumour prognosis, and response to therapy.

**Objectives:**

We assessed the application by immunohistochemistry method (IHC) for detection of schistosomal antigen in bladder cancer tissue samples to differentiate UC associated with or without schistosomiasis. Urothelial carcinoma stage, grade, and progression were correlated with the density, intensity, and index of schistosomal antigen expression. Follow up was done for 2-5 years.

**Design and participants:**

Archival tissue samples of 575 patients were studied: 515 urothelial carcinoma, 30 patients with SCC associated with schistosomiasis, and a control group of 30 patients without schistosomiasis.

**Measurements:**

Expression of schistosomal antigen in tissue was done by IHC using monoclonal antibodies (MAbs) against schistosomal antigens (SA). Correlation of intensity of antigen expression to clinical and pathological data was analysed.

**Results and limitations:**

We identified 3 parameters of antigen expression: density, intensity and index with 4 grades for each. SCC-group was 100% positive. UC was positive in 61.4% distributed as follows: Ta: 37.5%, T1: 62%, and muscle invasive T2-4 were 64%. Upstaging, metastases and recurrence were correlated with high index in T1 and T2-4 tumours.

**Conclusion:**

Urothelial carcinoma associated with schistosomiasis was defined by the positive expression of schistosomal antigens in tissues detected by lHC using MAbs against schistosomal haematobium. Upstaging and progression of T1 and T2-4 were correlated with high density, intensity, and index of antigen expression. Non-schistosomal UC had negative expression for schistosomal antigen, which was detected in 36.5% of cases. These results would be of significance in differentiating schistosomal from non-schistosomal bladder cancer of UC and would predict the prognosis in T1, T2-3 tumours. Implementation of IHC using MAbs against SA in UC would help in planning the proper therapy. Schistosomiasis should be considered as an oncogene for UC in endemic areas.

## Introduction

Urothelial carcinoma (UC) of the bladder is defined as the transitional cell carcinoma cell type (TCC). Squamous cell carcinoma (SCC) is a non-urothelial carcinoma. Scientific publications investigating bladder carcinoma in endemic areas with schistosomiasis amalgamated both UC and SCC as carcinoma associated with schistosomiasis, and consequently called it schistosomal or bilharzial bladder cancer [1,2].

Studies on tumour markers of UC associated with or without schistosomiasis from endemic areas showed controversial results compared to Western studies [3-6].

Publications dealing with schistosomal bladder lesions and carcinoma are dating back to 1960 and have been going on up to now. Their definition of schistosomal bladder cancer depends upon the clinical history of schistosomiasis, former urine analysis positive for schistosoma ova, history of received antibilharzial therapy, the finding of schistosoma ova in histopathology of tissue samples, tissue reaction of cystitis cystica, sandy patches or squamous metaplasia [4]. These criteria are not accurate and precise, additionally, the non-detection of schistosoma ova is not an exclusion possibility of a schistosomal association, as it is common that the examined tissue samples do not represent the whole bladder tissue, and the ova deposition is mostly focal and not diffuse.

The circulating schistosoma antigens would show the present status of patients but are not an indication of the past history and of the tissue reaction against schistosomiasis.

UC would develop in patients living in areas endemic with schistosomiasis. In spite of not having been infected with schistosomiasis, they were described as schistosomal bladder cancer. UC associated with or without schistosomiasis had previously not been defined. Its definition would assist in having a strategic therapeutic plan and would predict tumour progression.

Schistosomal antibody detection assay in serum is a sensitive procedure for the detection of circulating schistosomal antigens (CSA) by applying specific schistosoma haematobium (S.haematobium) monoclonal antibodies (MAbs) [7-11]. Schistosomal antigens in the bladder tissue would induce oncogenic cascades leading to UC [12,13].

Our aim was to establish a definition of UC associated with schistosomiasis by using the immunohistochemical technique of applying MAbs prepared against S.haematobium soluble egg antigens (SEA) on tissue samples of patients with UC and SCC and of control groups.

## Materials and methods

### Patients

The study included 575 patients. Their archival clinical data and tissue samples were collected and analysed in multi centric hospital and private clinics, part of the data being MSc theses. Follow-up of cases ranged from 2 to 5 years.

a) Urothelial Carcinoma

Enrolled in the study were 515 patients. The paraffin embedded blocks of tissue samples stained with hematoxylin and eosin were studied by light microscopy. Examination included’ archival samples since the first diagnosis. This studied group included 40 patients with Ta, 50 patients with T1, and 425 patients with muscle invasive of T2-4.

b) Squamous Cell Carcinoma

Thirty patients with SCC were included representing the positive reference. Histopathological examination showed presence of S.haematobium ova with schistosomal tissue reaction of cystitis cystica, sandy patches and squamous metaplasia.

c) Control Group

Twenty five patients having benign prostatic hyperplasia (BPH) underwent TURP. The covering urothelium was processed as control. 5 female patients diagnosed with interstitial cystitis (IC) had bladder biopsy. Part of the biopsy was used as control. Both series were schistosoma negative by detection of CSA in serum by the use of anti-S.haematobium Mabs, no history of Bilharziasis and no Bilharzial tissue reaction.

### Ethical agreement and Consent

This study was approved by the internal review board of Theodor Bilharz Research Institute, Cairo- Egypt for the use of the archival material and data. Written informed consent was obtained from patients for the use of their data and publish the results of the study, and any accompanying images.

### Monoclonal Antibodies against S.haematobium (MAbs anti-S.haematobium)

MAbs were prepared according to Cianfrigila [14]. Spleen cells from eight weeks old female BALB/C mice immunized with S.haematobium soluble antigens (SEA) were fused with non-secreting murine myloma cells (P3x63 Ag.8). Methodology of Galfre [15] was applied, fusion was performed in presence of 43% polyethylene glycol. Hybridomas were screened for antibodies against S.haematobium SEA antibodies by ELISA. Hybrids that were highly reactive with SEA were cloned by the limiting dilution method. Isotype analysis of MAbs was done using the mouse-hybridoma subtyping kits. Hybridoma cells were injected intraperitoneally into BALB/C mice for ascites production. MAbs 2D/11C and 10B/2C (IgGI) were purified by caprylic acid treatment followed by ion exchange chromatography. These MAbs are used to detect S. haematobium antigens in samples from 575 patients.

### Immunohistochemical Methods (IHM)

Indirect IHC on paraffin sections were applied according to Maranchie [16]. The paraffin sections were dewaxed, rehydrated, washed with phosphate buffered saline, incubated with 3% hydrogen peroxide to block endogenous peroxidase, washed again with phosphate buffer solution. The sections were boiled for 5 minutes in 10 mmol/l citrate buffer solution (pH 6.0) for antigen retrieval; sections were cooled and rinsed in Phosphate buffer solution. MAbs of anti S.haematobium SEA were incubated for 60 minutes at room temperature. Secondary antibody was applied followed by peroxidase-conjugated steptavidin. The demonstration of binding sites with peroxidase reaction was achieved with 3,3-diaminobenzidine tetrahydrochloride. The negative control estimation was performed by omitting the use of pimary antibodies.

### Interpretation of S. haematobium Antigen Expression in Tissues

Using IHC, the presence of S.haematobium antigens in tissues was detected by applying Mabs anti S.haematobium in paraffin embedded tissue samples of 575 patients.

a) Negative Control

The negative detection was referred to in the negative finding of the control groups of BPH and IC when applying the MAbs on a tissue section and on another section omitting its application respectively. Both techniques showed no expression of schistosoma antigen. This conclusion is described as negative or 0%.

b) Positive Control

Thirty patients who had SCC and presence of schistosoma ova in their tissue samples, chronic schistosomal lesions of cystitis cystica, sandy patches as well as squamous metaplasia were the positive control. They showed positive expression of tissue against S.haematobium antigens with high density and strong intensity and were considered to be the highest level of positivity of 100%.

c) Density of Schistosomal Antigen Expression

Percentage of positively stained cells in one microscopic field is referred to as density. A number of positively stained cells were recorded in five consecutive fields with the highest expression. The percentage was calculated from the mean. Degrees of density of schistosomal antigen expression were recorded as: negative (0%); one + (1% – 25%); two ++ (26% - 50%); three+++ (51% - 100%). Another nomenclature was: negative, low, moderate, high density.

d) Intensity of Schistosomal Antigen Expression

Intensity describes the deepness of colour intensity in the stained cells. The intensity of staining represents the body reaction towards schistosomal antigen. It is evaluated relative to the normal urothelial cell expression in normal control. Positive expression of S. haematobium antigen appears as a brown colour. Degrees in intensity of schistosomal antigen expression were recorded as: negative (0%); light intensity: one + (1% – 25%); moderate intensity: two ++ (26% - 50%); strong intensity: three+++ (51% - 100%). Another nomenclature was: negative, light, moderate, strong intensity.

e) Index of Schistosomal Antigen Expression

Index is referred to the sum of density and intensity of schistosomal antigen expression for each case. It is the mean of density and intensity of the positive antigen expression. The index was calculated for each case independently. Four ranges of indices were identified. 0: negative; low index: 1% - 25%; medium index: 25% - 50%; high index: 51% - 100%.

## Results

### Characteristics of the studied groups

The study included 575 patients. 30 cases were negative control as described in (Table 1). High positive reference was in 30 cases of SCC due to schistosomiasis that was proved by light microscopy and IHC methods, in addition to clinical history thse cases were the positive control. Urothelial carcinoma patients were 515, who had had different stages of UC of Ta, T1 and T2-4. Age and sex distribution of the 575 cases are elaborated in Table 1.

**Table 1:** Characterization of studied groups, 575 patients

**Diagnosis**	**Number**	**Age range (mean)**	**Sex**		**Total number**
			**Male**	**Female**	
Control - PBH	25	60-90 (73)	25	0	30
Control - IC	5	40-60 (45)	0	5	
Squamous Cell Carcinoma	30	35-65 (57)	27	3	30
Urothelial Carcinoma Ta	40	15-70 (55)	33	7	515
Urothelial Carcinoma T1	50	40-65 (56)	35	15	
Urothelial Carcinoma T2-4	425	45-74 (60)	381	54	

### Density of schistosomal antigen expression in urothelial carcinoma

The distribution of density in all studied groups was summarised in (Table 2).

**Table 2:** Density of schistosomal antigen expression in different studied groups (580 patients)

**Diagnosis**	**No. of cases**	**No. of positive cases**	**% of positive cases**	**Density of schistosomal antigen expression in tissue**			
**Not detected**	**Low**	**Moderate**	**High**
				**0% 0**	**1%-25%**	**26%-50%**	**51%-100%**
Control BPH	25	0	0%	25 (100%)	0	0	0
Control IC	5	0	0%	5 (100%)	0	0	0
SCC	30	30	100%	0 (0%)	0	8 (26.7%)	22 (73.3%)
Urothelial Carcinoma Ta	40	15	37.5%	25 (62.5%)	12 (80%)	3 (20.0%)	0 (0%)
Urothelial Carcinoma T1	50	31	62%	19 (38.0%)	14 (45.2%)	9 (29.0%)	8 (25.8%)
Urothelial Carcinoma T2-4	425	270	63.5%	155 (36.5%)	57 (21.1%)	120 (44.4%)	93 (34.5%)

Comments: 1- SCC was 100% positive for density of antigen expression.

2- The number and percentage of degrees of density of antigen expression were calculated in positive cases only.

3- Total number of positive cases in urothelial carcinoma were 316 out of 515 (61.5%).

#### Negative control groups

Twenty five men with BPH and five women with IC showed negative for expression of schistosomal antigen in urinary bladder tissue samples, and were negative for density expression.

#### Positive control group

**Figure 1:**
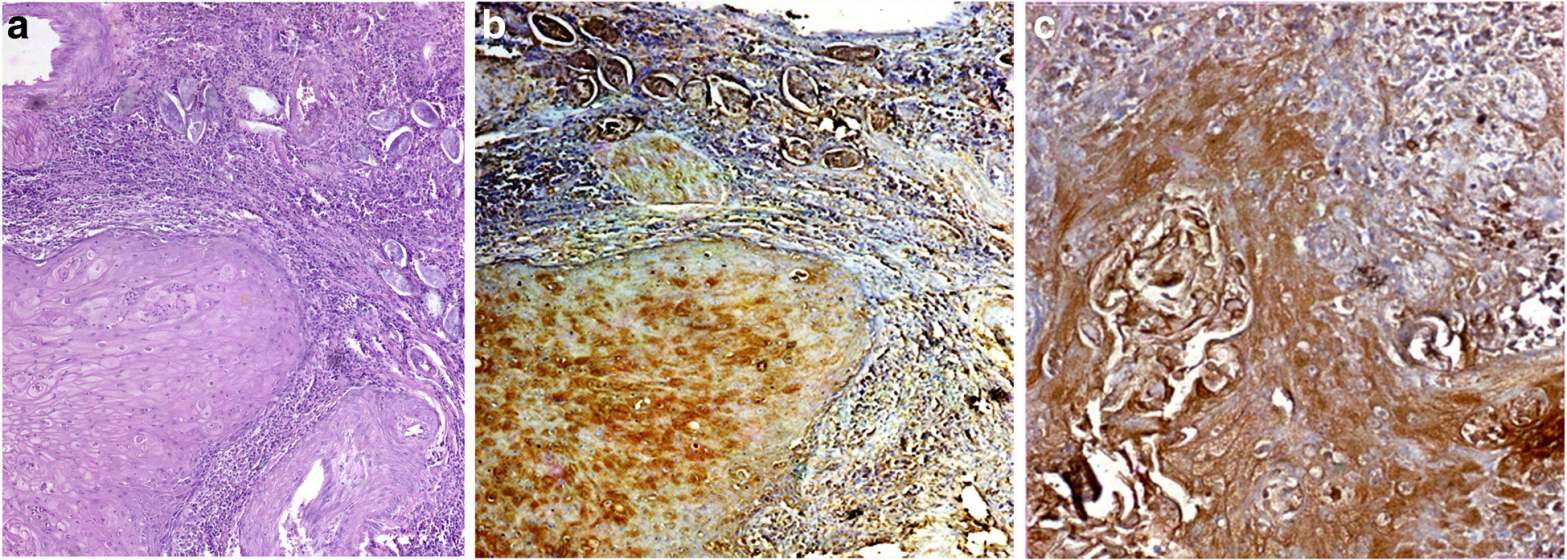
**Positive schistosomal antigen expression in squamous cell carcinoma group a.** Shows well differentiated SCC with schistosoma ova deposition. Hematoxylin and eosin stain , with omitting the monoclonal antibodies against S. haematobium did not show the antigen reaction. Original magnification × 200. **b**. Shows SCC with schistosoma ova deposition. Schistosomal antigen expression was positive with high intensity, density and index. Original magnification × 200. **c**. Shows SCC with high positive expression of Schistosomal antigen on application of Mab anti-S.Haematobium. Original magnification × 200.

Thirty patients with SCC were highly positive for schistosomal antigen expression, all cases were 100% over expressed. High density was in 73.3%, moderate density in 26.7%., there were neither negative nor low density cases (Figure 1a,b,c).

#### Urothelial carcinoma group

**Figure 2:**
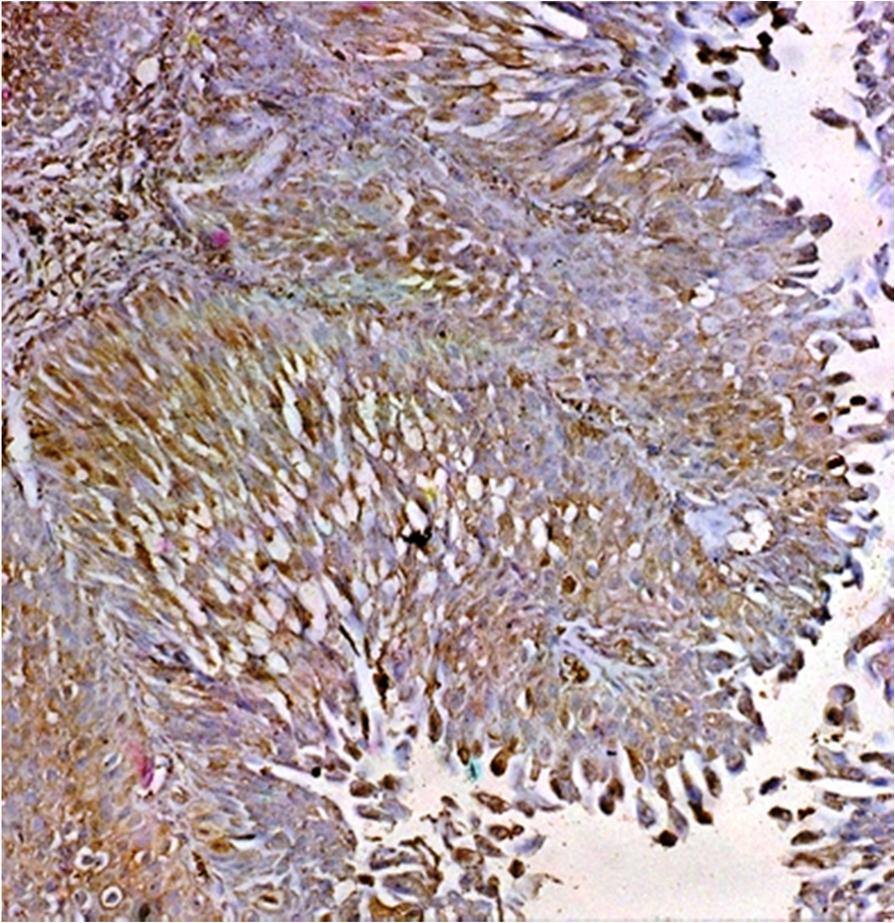
**Shows T1 urothelial carcinoma with positive schistosomal antigen expression with moderate intensity and density.** Original magnification × 200.

**Figure 3:**
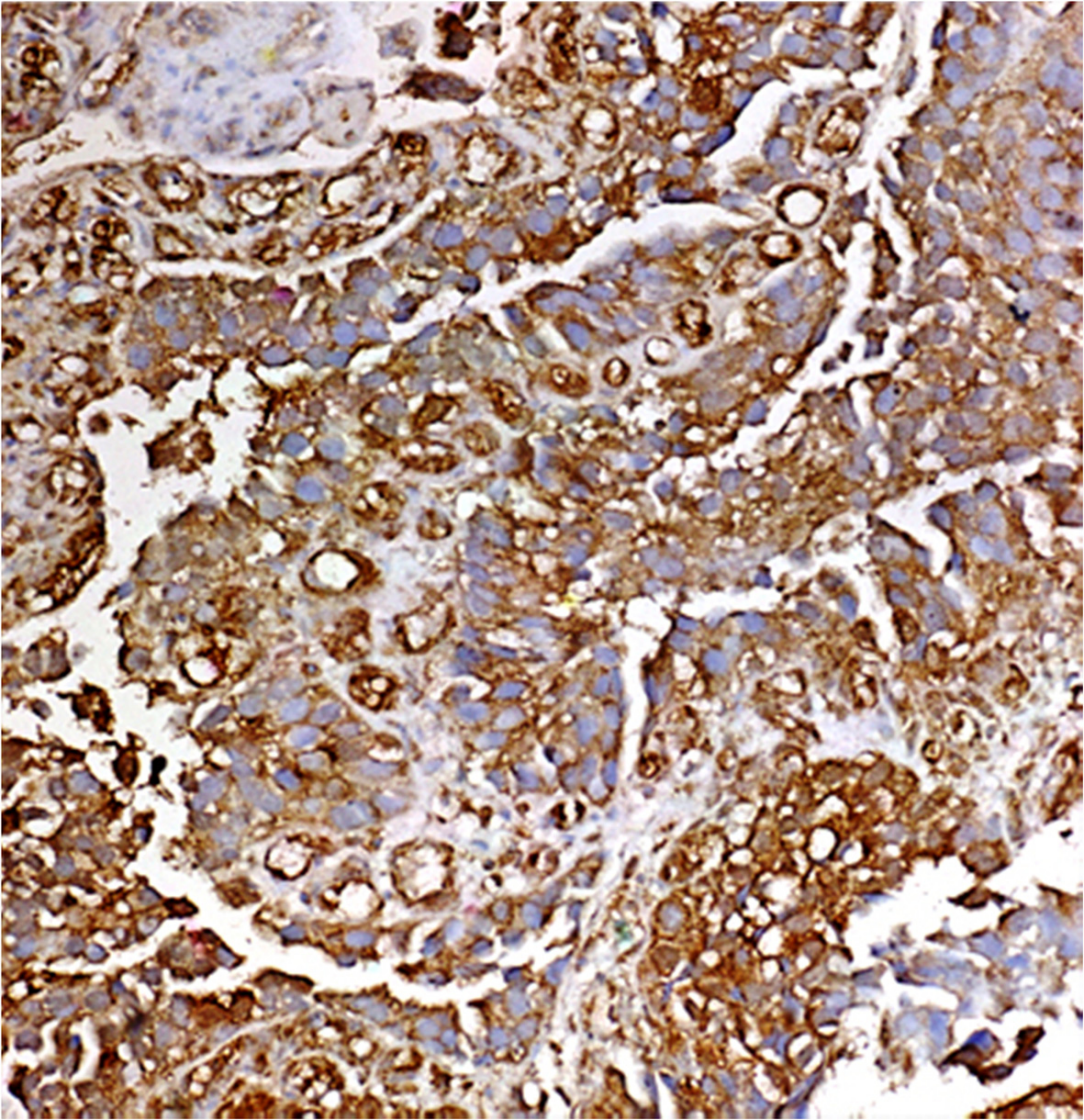
**Shows muscle invasive urothelial carcinoma without shistosoma ova deposition or schistosomal tissue reaction.** Schistosomal antigen expression was positive with high intensity, density, and index. Original magnification × 200.

Evaluated were 515 cases of UC. Ta tumours were 40 patients of whom 25 showed negative expression (62.5%). 15 were positive (37.5%). Low density was in 80%; moderate density in 20%.T1 tumours were 50 cases. Negative expression was in 38%, positive cases were 31 (62%) in which the distribution of density was high in 25.8%, medium in 29%, and low in 45.2% (Figure 2).T2-4 was evaluated in 425 cases of which 155 were negative (36.5%) (Figure 3). In the 270 positive cases (63.5%), the density was high: 34.5%, moderate: 44.5%, and low: 21%.

### Intensity of schistosomal antigen expression

The intensity represented the magnitude of tissue reaction to the schistosomal antigen. Intensity of antigen expression in all studied groups was elaborated in (Table 3). Intensity was negative in the control groups. The positive group of SCC showed strong expression in 86.7%, while 13.3% had moderate intensity. Light intensity was not recorded.

**Table 3:** Intensity of schistosomal antigen expression in different studied groups (580 patients)

**Diagnosis**	**No. of cases**	**No. of positive cases**	**% of positive cases**	**Intensity of schIstosomal antigen expression in tissue**			
				**No antigen**	**Light**	**Moderate**	**Strong**
				**0% 0**	**1%-25%**	**26%-50%**	**51%-100%**
Control BPH	**25**	**0**	**0%**	25 (100%)	**0**	**0**	**0**
Control IC	**5**	**0**	**0%**	5 (100%)	**0**	**0**	**0**
SCC	**30**	**30**	**100%**	0 (0%)	0	4 (13.3%)	26 (86.7%)
Urothelial Carcinoma Ta	40	15	37.5%	25 (62.5%)	10 (66.7%)	5 (33.3%)	0 (0%)
Urothelial Carcinoma T1	50	31	62%	19 (38.0%)	5 (16.2%)	16 (51.6%)	10 (32.2%)
Urothelial Carcinoma T2-4	425	270	63.5%	155 (36.5%)	64 (23.7%)	130 (48.1%)	76 (28.2%)

Comments: 1- SCC was 100% positive for intensity of antigen expression.

2- The number and percentage of degrees of intensity of antigen expression were calculated in positive cases only.

3- Total number of positive cases in urothelial carcinoma were 316 out of 515 (61.5%).

Ta tumors showed positive intensity in 37.5%, light intensity in 66.7% and moderate intensity in 33.3%.

T1 tumors showed strong intensity in 32.2%, moderate in 51.6%, and light intensity in 16.2% of cases.

T2-4 tumors were positive in 270/425 (63.5%) cases, negative cases were 36.5%. 24.7% of positive cases had light intensity, 48% moderate, and 28% strong intensity.

### Index of schistosomal antigen expression

Index of expression of antigen expression in all studied groups was elaborated in (Table 4).

**Table 4:** Index^* ^**of schistosomal antigen expression in different studied groups (580 patients)**

**Diagnosis**	**No. of cases**	**No. of positive cases**	**% of positive cases**	**Index of schIstosomal antigen expression in tissue**			
				**Index 0**	**Low index**	**Medium index**	**High index**
				**0% 0**	**1%-25%**	**26%-50%**	**51%-100%**
Control BPH	25	0	0%	25 (100%)	0	0	0
Control IC	5	0	0%	5 (100%)	0	0	0
SCC	30	30	100%	0 (0%)	0	4 (13.3%)	26 (86.7%)
Urothelial Carcinoma Ta	40	15	37.5%	25 (62.5%)	13 (86.7%)	2 (13.3%)	0 (0%)
Urothelial Carcinoma T1	50	31	62%	19 (38.0%)	6 (19.4%)	16 (51.6%)	9 (29%)
Urothelial Carcinoma T2-4	425	270	63.5%	155 (36.5%)	33 (12.2%)	110 (40.8%)	127 (47%)

*Index = (Density + Intensity)/2.

Significance of index of expression of schistosomal antigen and tumor progression was elaborated in (Table 5).

**Table 5:** Correlation between index of schistosomal antigen expression in urothelial carcinoma positive cases and tumor progression in 5 years follow up

**Stage of urothelial carcinoma**	**No. of positive index cases**	**Low index**		**Medium index**		**High index**	
		**No. of cases**	**Progression**	**No. of cases**	**Progression**	**No. of cases**	**Progression**
Urothelial Carcinoma Ta	15	12 (80%)	0 (0%)	3 (20%)	0 (0%)	0 (0%)	0 (0%)
Urothelial Carcinoma T1	31	6 (19.4%)	0 (0%)	16 (51.6)	13/16 (81.2%)	9 (29%)	9/9 (100%)
Urothelial Carcinoma T2-4	270	36 (13.3%)	7/36 (19.4%)	119 (44.1%)	90/119 (75.6%)	115 (42.6%)	109/127 (85.8%)

Comments: 1-Progression is estimated for each degree of the index.

2-Tumour progression indicates upstaging in T1 tumors.

3-Tumour progression indicates local recurrence and metastases in T2-4 after definitive therapy.

Index was recorded for every patient and correlated with the tumour progression in T1 and recurrence in T2-4.

Ta positive cases had low index in 86.7%, medium index in 13.3%, high index was not found. In 5 years follow-up, no case had progression.

T1 positive cases were 19.4% with low index, with no upstaging. 16/31 cases of T1 tumour had a medium index of 51.5% with 81% having upstaging to muscle invasion in five years follow-up. T1 with high index were 29%, all of them had upstaging.

T2-4 were 270 out of 425 with 63.5% positive index: 12.2% low index, 40.8% medium and 47% high index. Progression was found in 21.2% of the low, 81.8% of the medium and 95% of the high index group.

The SCC tumours had a high index in 68.7% and a medium index in 13.3%.

## Discussion

The urothelial carcinoma associated with schistosomiasis has not been defined or standardised in literature [1-3]. For the definition of schistosomal UC, we introduced the use of an immunohistochemical method to evaluate the expression of schistosomal antigens in tissue using MAbs anti-S.haematobium in 575 patients. 30 were SCC and served as positive control, 30 with BPH and IC as negative control. 515 were UC of Ta, T1, T2-4.

Our results showed that 61.4% of the 515 urothelial carcinoma cases were positive while 38.6% were negative. This finding differentiated between the schistosomal and non-schistosomal urothelial carcinoma.

The density of schistosomal antigen expression denotes the antigen load in tissue that was found high in T1, T2-4, contrary to low density in Ta tumours.

Intensity of schistosomal antigen expression represented the severity of tissue and cellular reaction against schistosomal antigen. It was found to be strong and moderate in T1, T2-4, while low in Ta tumours.

Our study indicated that high density and strong intensity were associated with invasive stages where Ta had a low density and light intensity.

The impact of schistosomiasis on the urinary bladder had been considered to be a promoter for carcinoma [12,13] which was confirmed in our clinical study. The presence of strong intensity and high density in high stages of tumours denoted the schistosomal antigen tumour genesis in urothelial carcinoma associated with schistosomiasis [12].

Our study indicated that index of schistosomal antigen was a predictive marker for progression in schistosomal urothelial carcinoma, for upstaging, recurrence, and/or metastases. Our results indicate that for the achievement of proper therapy and prediction of prognosis of UC associated with schistosomiasis, detection of schistosomal antigen parameters is crucial. The study showed that the strong and high antigen expression defined by index of antigen expression has an impact on tumour upstaging and progression, where high index indicated tumour upstaging, progression, and aggressiveness.

## Conclusion

Urothelial carcinoma associated with schistosomiasis is defined by the positive expression of schistosomal antigens in tissues that were detected by IHC using MABs against S. haematobium. In our study, we described three parameters of tissue expression: Density and intensity with 4 grades, and index with 3 grades of low, medium, and high. They denoted the tissue reaction to Schistosomal antigens and its tumourogenesis. High schistosomal antigen expression was statistically significant with higher tumour stage, high index was significant for upstaging in T1 and for progression to recurrence and metastases in T2-3 tumors. Low antigen expression with low density, intensity and zero index were correlated with low risk tumors of Ta. Negative expression of antigens was found in 36.5% of patients with UC who should be excluded from the group of schistosomal bladder cancer. High index of schistosomal antigens demands a close surveillance and early radical intervention.

## Abbreviations

UC: Urothelial carcinoma; TCC: Transitional cell carcinoma; SCC: Squamous cell carcinoma; MAbs: Monoclonal antibodies; SEA: Schistosomal egg antigen; IHC: Immunohistochemical method; SA: Schisosomal antigen; CEA: Circulating egg antigen; BPH: Benign prostatic hyperplasia; IC: Interstitial cystitis.

## Competing interests

The authors declare that they have no competing interests, and have nothing to disclose.

## Authors’ contributions

MW Made the design and concept of the study, acquisition of data, analysis and interpretation of data, drafting the manuscript, and performed the statistical analysis. AZ Made the design and concept of the study, acquisition of data, analysis and interpretation of data. OH participated in the design of the study, acquisition of data, analysis and interpretation of data. MA Participated in the design and concept of the study. HB Participated in the design and concept of the study. HE Participated in the drafting of the manuscript, acquisition of data, participated in the design of the study. MI Participated in design of the study. AE Participated in the drafting of the manuscript, acquisition of data. AM Participated drafted the manuscript, participated in the design of the study. All authors read and approved the final manuscript.

## References

[B1] Shaker OG Hammam OA El Ganzoury H Wishahi MM Molecular markers and bladder carcinoma: schistosomal and non-schistosomal Clin Biochem 2011 44 237 240 20934419 10.1016/j.clinbiochem.2010.09.028

[B2] Shaker OG Hammam O Wishahi M Possible role of telomerase and sFas in pathogenesis of various bladder lesions associated with schistosomiasis Clin Biochem 2009 42 884 888 10.1016/j.clinbiochem.2008.12.025 19272297

[B3] Rosin MP Anwar W Chromosomal damage in urothelial cells from Egyptians with chronic schistosoma haematobium infection Int J Cancer 1998 50 539 543 1537619 10.1002/ijc.2910500407

[B4] Haital A Posch B El-Baz M Bilharzial related, organ confined, muscle invasive bladder cancer: prognostic value of apoptosis markers, proliferation markers, P53, E-cadherin, epidermal growth factor receptor and C-erb-B-2 J Urol 2001 165 1481 1487 11342901

[B5] Adulamir AS Hfidh RR Kadh A Tumor markers of bladder cancer: the schistosomal bladder tumors versus non-schistosomal bladder tumors J Exp Clin Cancer Res 2009 25 27 28 10.1186/1756-9966-28-27 PMC2650688 19243595

[B6] Ghoneim MA Adel-Latif M El Mekresk M Radical cystectomy for carcinoma of the bladder: 2,720 consecutive cases 5 years later J Urol 2008 180 121 127 18485392 10.1016/j.juro.2008.03.024

[B7] Tijssen P Kurstak P Highly efficient and simple methods for the preparation of peroxidase and active peroxidase antibody conjugates for enzyme immuno-assays Anal Biochem 1984 136 451 458 6372541 10.1016/0003-2697(84)90243-4

[B8] Salah F Demerdash Z Shaker Z A monoclonal antibody against schistosoma haematobium soluble egg antigen: efficacy for diagnosis and monitoring for cure of S.haematobium infection Parasitol Res 2000 86 74 80 10669141 10.1007/s004360050014

[B9] Nibbeling HA Kahama AI Van Zeyl RJ D: Use of monoclonal antibodies prepared against schistosoma haematobium circulating egg antigens in urine Am J Trop Med Hyg 1998 85 453 550 10.4269/ajtmh.1998.58.543 9598438

[B10] Salah F El Bassiouny A Rabia I Human schistosomiasis haematobium: effective diagnosis of active infection using a pair of monoclonal antibodies against soluble egg antigen Parasitol Res 2006 99 528 533 16633832 10.1007/s00436-005-0016-8

[B11] Mohamed SH Demerdash ZA Shaker ZA Production and characterization of monoclonal antibody reactive with repetitive of concanavalin. A bound glycoprotein fraction of S.mansoni soluble egg antigen J Egypt Med Assoc 1995 78 37 49

[B12] Botelho MC Machado JC Costa JU Schistosoma haematobium and bladder cancer: What lies beneath? Virulence 2010 2 84 87 21178421 10.4161/viru.1.2.10487

[B13] El Awady MK Gad YZ Wan Y Eassawwi M Schistosoma hematobium soluble antigens in due proliferaton of urothelial and endothelial cells World J Urol 2001 19 263 269 11550787 10.1007/s003450100217

[B14] Cianfriglia M Armellini D Massone A Mariani M Simple immunization protocol for high frequency production of soluble antigen-specific hybridomas Hybridoma 1983 2 451 457 6678805 10.1089/hyb.1983.2.451

[B15] Galfrè G Milstein C Preparation of monoclonal antibodies. Strategies and procedures Methods Enzymol 1981 73 3 46 7300683 10.1016/0076-6879(81)73054-4

[B16] Maranchie JK Bouyounes BT Zhang PL O’Donnell MA Summerhayes IC De Wolf WC Clinical and pathological characteristics of micropapillary transitional cell carcinoma: a highly aggressive variant J Urol 2000 163 748 751 10687969

